# Identifying neutrophil-associated subtypes in ulcerative colitis and confirming neutrophils promote colitis-associated colorectal cancer

**DOI:** 10.3389/fimmu.2023.1095098

**Published:** 2023-02-10

**Authors:** Chen Zhang, Jiantao Zhang, Yanli Zhang, Zian Song, Jing Bian, Huanfa Yi, Zhanchuan Ma

**Affiliations:** ^1^ Colorectal & Anal Surgery Department, General Surgery Center, First Hospital of Jilin University, Changchun, Jilin, China; ^2^ Central Laboratory, First Hospital of Jilin University, Changchun, Jilin, China; ^3^ Echocardiography Department, First Hospital of Jilin University, Changchun, Jilin, China; ^4^ Department of Respiratory Medicine, First Affiliated Hospital of Jilin University, Changchun, Jilin, China

**Keywords:** ulcerative colitis, colitis-associated colorectal cancer, immune cell infiltration, biomarker, neutrophil

## Abstract

**Background:**

Ulcerative colitis (UC) is a chronic inflammatory disease of the intestinal mucosa, the incidence of which has increased worldwide. There is still a lack of clear understanding of the pathogenesis of ulcerative colitis that ultimately leads to colitis-associated colorectal cancer.

**Method:**

We download UC transcriptome data from the GEO database and pass the limma package in order to identify differentially expressed genes. Gene Set Enrichment Analysis (GSEA) was used to identify potential biological pathways. We identified immune cells associated with UC by CIBERSORT and Weighted co-expression network analysis (WGCNA). We used validation cohorts and mouse models to verify the expression of the hub genes and the role of neutrophils.

**Result:**

We identified 65 differentially expressed genes in UC samples and healthy controls. GSEA, KEGG, and GO analyses displayed that DEGs were enriched in immune-related pathways. CIBERSORT analysis revealed increased infiltration of neutrophils in UC tissues. The red module, obtained by WGCNA analysis, was considered to be the most relevant module for neutrophils.Based on neutrophil-associated differentially expressed genes, UC patients were classified into two subtypes of neutrophil infiltration. We discovered that the highly neutrophil-infiltrated subtype B of UC patients had a higher risk of developing CAC. Five genes were identified as biomarkers by searching for DEGs between distinct subtypes. Finally, using the mouse model, we determined the expression of these five genes in the control, DSS, and AOM/DSS groups. The degree of neutrophil infiltration in mice and the percentage of MPO and pSTAT3 expression in neutrophils were analyzed by flow cytometry. In the AOM/DSS model, MPO and pSTAT3 expressions were significantly increased.

**Conclusions:**

These findings suggested neutrophils might promote the conversion of UC into CAC. These findings improve our understanding of the pathogenesis of CAC and provide new and more effective insights into the prevention and treatment of CAC.

## Introduction

Ulcerative colitis (UC) is a chronic inflammatory disease affecting the intestine that begins in the rectum and could progress to the entire colon. Its clinical symptoms are mainly characterized by persistent or recurrent diarrhea, mucopurulent, and bloody stools, and may be accompanied by various degrees of systemic symptoms (1, 2). Ulcerative colitis is considered a chronic immune-mediated progressive disease with a rapidly increasing global incidence (3). UC causes a variety of intestinal diseases and patients with a long course eventually develop inflammation-associated malignancies, thus affecting their quality of life (4). Inflammation levels, duration, and severity of UC are also linked to an increased risk of colitis-associated colorectal cancer (CAC) (5–7). The pathophysiology of UC is complex and not fully understood. A defective intestinal epithelial barrier, environmental factors, genetic susceptibility, and a dysfunctional immune system are all thought to be involved in UC pathogenesis (8–10). For chronic inflammation, epithelial cells undergo a transition from injured and regenerating but non-dysplastic tissue to the progressive neoplastic epithelium, with molecular and genetic changes accompanying this histological progression from low-grade dysplasia (LGD) to high-grade dysplasia (HGD) and ultimately to adenocarcinoma (11–14).

Neutrophils play a primary function in the development and maintenance of intestinal inflammation. Chemokines and reactive oxygen species (ROS) are produced, the epithelial barrier is disrupted, other immune cells are recruited and activated, and redox-sensitive inflammatory pathways are activated (15). Studies have shown that large numbers of neutrophils infiltrate the UC colonic mucosa, releasing serine proteases, matrix metalloproteinases, and myeloperoxidases through the production of reactive oxygen species, directly causing tissue damage and producing the typical crypt abscesses (16, 17). Neutrophils could also exacerbate intestinal inflammation and cause malignancies by producing neutrophil extracellular traps (NETs) (18). NETs are reticular structures that induce the formation of NETs through the conversion of arginine residues to citrulline dependent on peptidyl arginine deiminase type IV (PAD4) (19–21). Myeloperoxidase (MPO), a heme peroxidase that is mostly kept in the nitrogenophilic granules of neutrophils and produced following the activation of neutrophils (22). High concentrations of MPO in NETs also induce oxidative stress in epithelial cells, exacerbating DNA damage and mutations (23). NETs could cause metastatic tumors that exacerbate cancer and NETs could aggravate autoimmune diseases such as rheumatoid arthritis and systemic lupus erythematosus (24, 25). In addition, increased NETs formation is associated with COVID-19-associated ARDS and is a potential biomarker of disease severity and clinical outcome (26).

In this study, we analyzed the correlation between neutrophils and ulcerative colitis using the WGCNA approach and found 24 neutrophils-associated differentially expressed genes (NADEGs). Based on the expression of NADEGs, UC patients were classified into two subtypes. The differences in severity and immune cell infiltration between patients of different subtypes were also assessed. We identified five genes as biomarkers for colitis conversion to colorectal cancer based on the differential genes between subtypes. We then validated the signature gene in the DSS and AOM/DSS mouse models. Flow cytometry analysis also showed that neutrophils increased with the progression of colitis. As a result of these findings, new insights into the diagnosis and treatment of colitis and colitis-associated colorectal cancer will be gained.

## Materials and methods

### Data collection and processing

**Figure S1** displayed the flowchart for the study. Gene expression profiles of ulcerative colitis were retrieved from the GEO database (http://www.ncbi.nlm.nih.gov/geo/). A total of 803 RNA-seq samples were downloaded from datasets GSE87473, GSE92415, and GSE206285, including untreated 743 UC samples and 60 healthy control samples. It should be noticed that though 364 patients with UC were treated with ustekinumab (data extracted from database GSE206285), RNA extraction and microarray analysis of biopsy samples collected when the data set was uploaded at baseline. Baseline is the period of time in a clinical study when a patient has been screened to for the study but has not yet started medication. To convert the probe expression matrix to the gene expression matrix, we used the platform annotation file. The average of multiple probes corresponding to the same gene is calculated. To remove the batch effect between datasets and merge them, we used the “ComBat” function in the SVA package. Equally, the CAC dataset was obtained from dataset GSE37283. For validation, GSE53306 and GSE16879 datasets were also downloaded and processed. The included datasets and baseline clinical characteristics of patients with UC were provided in **Supplementary Table S1**.

### Identification of DEGs and functional enrichment analysis

Based on RNA-seq data obtained from GEO (743 UC samples and 60 normal samples), we used the LIMMA package to identify differentially expressed genes (DEGs). Volcano and heatmap visualizations of the results were created using the ggplot2 package in R. DEGs were analyzed using Gene Ontology (GO) and Kyoto Encyclopedia of Genes and Genomes (KEGG) using the ClusterProfiler package in R. GO analyses were conducted for cellular components (CC), biological processes (BP), and molecular functions (MF).

### Immune cell infiltration analysis

To calculate immune cell infiltration and examine the disease immune microenvironment, we used the algorithm CIBERSORT and iterated 1000 times (27). Using the ggplot2 package, we visualized the results of immune cell infiltration. For visualization of immune cell infiltration correlations, Corrplot was used to calculate Spearman analysis correlations between immune cells.

### Identification of neutrophil-associated differentially expressed genes

The WGCNA package was used to identify genes associated with immune cell infiltration. The gene expression matrix outliers were filtered using hierarchical clustering. Based on the expression data, Pearson correlation coefficients were calculated between two genes to construct a similarity matrix. Using a soft threshold of 15, the similarity matrix was transformed into an adjacency matrix and then into a topology matrix with a topological overlap measure (TOM). Modules are identified using a dynamic pruning tree and clustered based on 1-TOM distance. Setting the merge threshold to 0.25 resulted in the identification of 14 modules. By intersecting DEGs with genes associated with neutrophils (red modules), we identified neutrophil-associated differentially expressed genes (NADEGs). A search for hub genes and functional analysis of NADEGs in Cytoscape (3.9.1) using Cytohubba and Cluego.

### Neutrophil-related subtype identification based on NADEGs

We performed an unsupervised cluster analysis using ConsensuClusterPlus to identify different neutrophil infiltrating subtypes. Functional annotation and GSVA were used to identify biological differences between subtypes. GSVA was performed on gene sets from the MSigDB database (C2.Cp.ke.v7.2) with statistical significance set at adj.P<0.05. To assess immune cell infiltration, ssGSEA (single sample gene set enrichment analysis) was performed. In addition, we used the ESTIMATION algorithm to assess the stromal and immune score in each sample. Additionally, we compared clinical characteristics (age and disease extent) between subtypes.

### Finding specific biomarkers for the conversion of colitis to CAC

Using the LIMMA package, the DEGs between various subtypes were found, and the criterion was | log2 FC | >0.585 with an adjusted p-value< 0.05. To test the predictive value of the identified biomarkers, we assessed the diagnostic validity of UC and control sample differentiation using the area under the ROC curve (AUC) values. Using Spearmen analysis, the relationship between the detected biomarkers and the quantity of invading immune cells was investigated.

### Construction of the mouse model

The experiments were conducted with 8-10 weeks male mice under specific pathogen-free conditions (SPF). The AOM/DSS model was constructed using C57BL/6 mice, which is well-established for replicating the immune response in colitis (28). All mice were purchased from Vital River (Beijing, China). The Animal Health Research Committee of the First Hospital of Jilin University approved all animal experiments. The mice were divided into three groups, control, DSS and AOM/DSS, with three mice in each group. AOM/DSS models were constructed as follows: mice were intraperitoneally injected with 10 mg/kg of AOM on the first day. A week later, mice were fed 2% DSS solution (40-50 KDa DSS (Sigma Aldrich) dissolved in water at a final concentration of 2%) for 5 consecutive days, and so on for 3 cycles. The mice in the DSS group were fed with 2% DSS for 5 consecutive days and were regularly fed water for the remaining days. After 6 weeks, mice were euthanized and colonic tissues were collected for hematoxylin and eosin (H&E) staining, flow cytometric analysis, and RT-qPCR.

### RNA extraction and RT-qPCR in tissue samples

Colon tissues were taken from different groups of mouse colons, and we extracted total RNA from the tissues using the Animal Total RNA Isolation kit (FOREGENE, China) and determined its purity and concentration. We used the Uni RT&qPCR kit (transgen, China) to reverse convert RNA to cDNA and performed RT-qPCR on an instrument (ABI QuantStudio 3, USA). We used β-actin as an internal reference and 2-ΔΔCt to calculate the relative gene expression levels and Graphpad 9.0 to visualize the data. A detailed list of primer sequences used for RT-qPCR is shown in **Table S2**.

### H&E staining

Tissues from the colon were embedded in paraffin after being treated with 4 percent paraformaldehyde (PFA) overnight. To prepare a glass slide for H&E staining, 5 um-thick pieces of the colon were cut out and set flat on it.

### Tissue preparation

Mice were sacrificed to obtain tissues and cells for detection. 5ml cold PBS was injected into the enterocele of mice, gently shaken the body, and collected PBS into 15ml centrifuge tubes to obtain peritoneal irrigation fluid (PIF). To obtain neutrophils in peripheral blood (PB), cells were separated by density gradient centrifugation (Mouse peripheral blood lymphocyte isolation kit, solarbio, China), and neutrophils were obtained after lysed red blood cells in the sedimentation. Peyer’s patches (PP) were carefully detached from the small intestine, and mesenteric lymph nodes (MLN) were carefully detached from the colon and placed in PBS. To collect lamina propria mononuclear cells (LPMC), the colon of mice was cut into small pieces (about 1mm3 in volume), mixed tissue pieces with digestion buffer (PBS containing 5 mM EDTA and penicillin/streptomycin) and shaken for 20 mins to remove intraepithelial lymphocytes. Then, the tissue pieces were digested with 1 mg/ml collagenase IV and 10 U/ml DNase I at 37°C for 1 h. All cells were obtained by passing the tissues through a 70 mm strainer.

### Flow cytometric analysis

5 * 105 cells were washed twice in FACS buffer and stained for CD45 Pacific Blue (S18009F), CD11b APC/Cyanine7 (M1/70), Ly 6g PerCP/Cyanine5.5 (1A8) for 30 mins. For intracellular stain, cells were treated with the transcription factor staining buffer set according to the manufacturer’s specification (eBioscience, USA). Then, cells were stained with the following antibodies: rabbit anti-mouse myeloperoxidase (Abcam, England), STAT3 Phospho (Tyr705) PE antibodies for 30 mins, then cells were washed with FACS buffer and stained with goat anti-rabbit IgG H&L Alexa Fluor^®^ 488 for 30 mins. Then, cells were washed twice for flow cytometric analysis. Antibodies for CD45, CD11b, Ly6g, and pSTAT3 were purchased from Biolegend. Antibodies for myeloperoxidase and goat anti-rabbit IgG H&L were purchased from Abcam. Flow cytometric analyses were conducted using an Arial flow cytometer (BD Biosciences). Data were evaluated using FlowJo software (Version 10; FlowJo).

### Statistical analysis

The statistical analyses were conducted using R (version 4.2.0). Continuous variables were compared between groups, and normally distributed variables were compared using the t-test. Diagnostic biomarkers were evaluated using ROC curve analysis. Spearman correlation was used to analyze the relationship between infiltrating immune cells and gene biomarkers. All statistical analyses were two-sided, and P < 0.05 was considered statistically significant.

## Result

### Identification and functional enrichment analysis of DEGs

**Figure 1:**
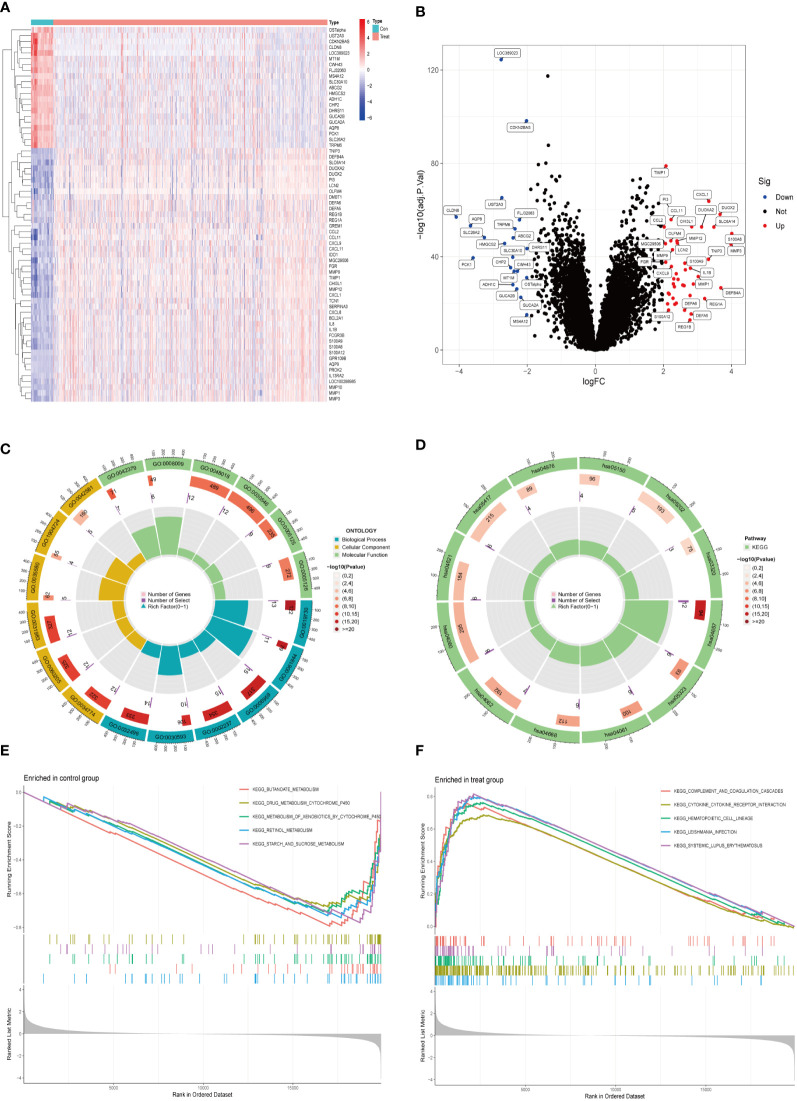
Identification of DEGs between UC and control samples. **(A)** The expression of 65 DEGs was visualized by heatmap. Red for upper regulation, blue for lower regulation **(B)** Volcano map of DEGs in UC compared to healthy controls. The significantly upregulated DEGs (log2FC>2, adj.P<0.05) were labeled in red, and the significantly downregulated DEGs (log2FC<2, adj.P<0.05) were labeled in blue. **(C, D)** GO and KEGG enrichment analyses of DEGs. The outermost circle was the GO number, the second circle represented the number of genes on the GO, and the third circle represents the number of genes enriched on the GO. The color represented the p-value of significant enrichment, the redder the color, the more significant the enrichment. **(E, F)** Results of GSEA pathway analysis in UC and healthy control samples.

We collected 743 UC patients and 60 healthy controls from the GSE87473, GSE92415, and GSE206285 datasets. Using the battle algorithm in the SVA package, the combined dataset was obtained by removing batch effects from the GEO dataset. DEGs between UC and healthy controls were analyzed by the LIMMA package (|LogFC|>2, p<0.05). There were found to be 65 DEGs in total, of which 22 had down-regulation and 44 had upregulation (**Table S3**). Heatmap and volcano plots illustrated these upregulated and downregulated genes (**Figures 1A, B**). The biological features of these DEGs were then ascertained by the GO analysis and KEGG analysis. GO functional enrichment analysis revealed that DEGs were significantly enriched in granulocyte chemotaxis, neutrophil chemotaxis, and neutrophil migration in the BP category. There was a significant enrichment in the cytoplasmic vesicle lumen and secretory granule lumen in the CC category. The MF terms were enriched with cytokine activity, chemokine receptor binding, and cytokine receptor binding (**Figure 1C** and **Table 1**). KEGG pathway analysis revealed regulated terms (FDR<0.05), including IL-17 signaling pathway, Cytokine-cytokine receptor interaction, NF-κB signaling pathways, and TNF signaling pathway (**Figure 1D** and **Table 2**). We also compared the respective significantly enriched pathways in the healthy control and UC cohorts. As shown in **Figures 1E, F**, immune-related pathways were enriched in the UC cohort compared to healthy controls. These results suggested that inflammation-related pathways are enriched in UC patients.

**Table 1:** GO enrichment results for 65 DEGs.

ONTOLOGY	ID	Description
BP	GO:0019730	antimicrobial humoral response
BP	GO:0061844	antimicrobial humoral immune response mediated by antimicrobial peptide
BP	GO:0006959	humoral immune response
BP	GO:0002237	response to molecule of bacterial origin
BP	GO:0030593	neutrophil chemotaxis
BP	GO:0032496	response to lipopolysaccharide
BP	GO:0071621	granulocyte chemotaxis
CC	GO:0034774	secretory granule lumen
CC	GO:0060205	cytoplasmic vesicle lumen
CC	GO:0031983	vesicle lumen
CC	GO:0035580	specific granule lumen
CC	GO:1904724	tertiary granule lumen
CC	GO:0042581	specific granule
CC	GO:0070820	tertiary granule
MF	GO:0042379	chemokine receptor binding
MF	GO:0008009	chemokine activity
MF	GO:0048018	receptor ligand activity
MF	GO:0030546	signaling receptor activator activity
MF	GO:0005125	cytokine activity
MF	GO:0005126	cytokine receptor binding
MF	GO:0045236	CXCR chemokine receptor binding

**Table 2:** KEGG enrichment results for 65 DEGs.

ID	Description
hsa04657	IL-17 signaling pathway
hsa05323	Rheumatoid arthritis
hsa04061	Viral protein interaction with cytokine and cytokine receptor
hsa04668	TNF signaling pathway
hsa04062	Chemokine signaling pathway
hsa04060	Cytokine-cytokine receptor interaction
hsa04621	NOD-like receptor signaling pathway

### Analysis of immune cell landscapes and identification of NADEGs

**Figure 2:**
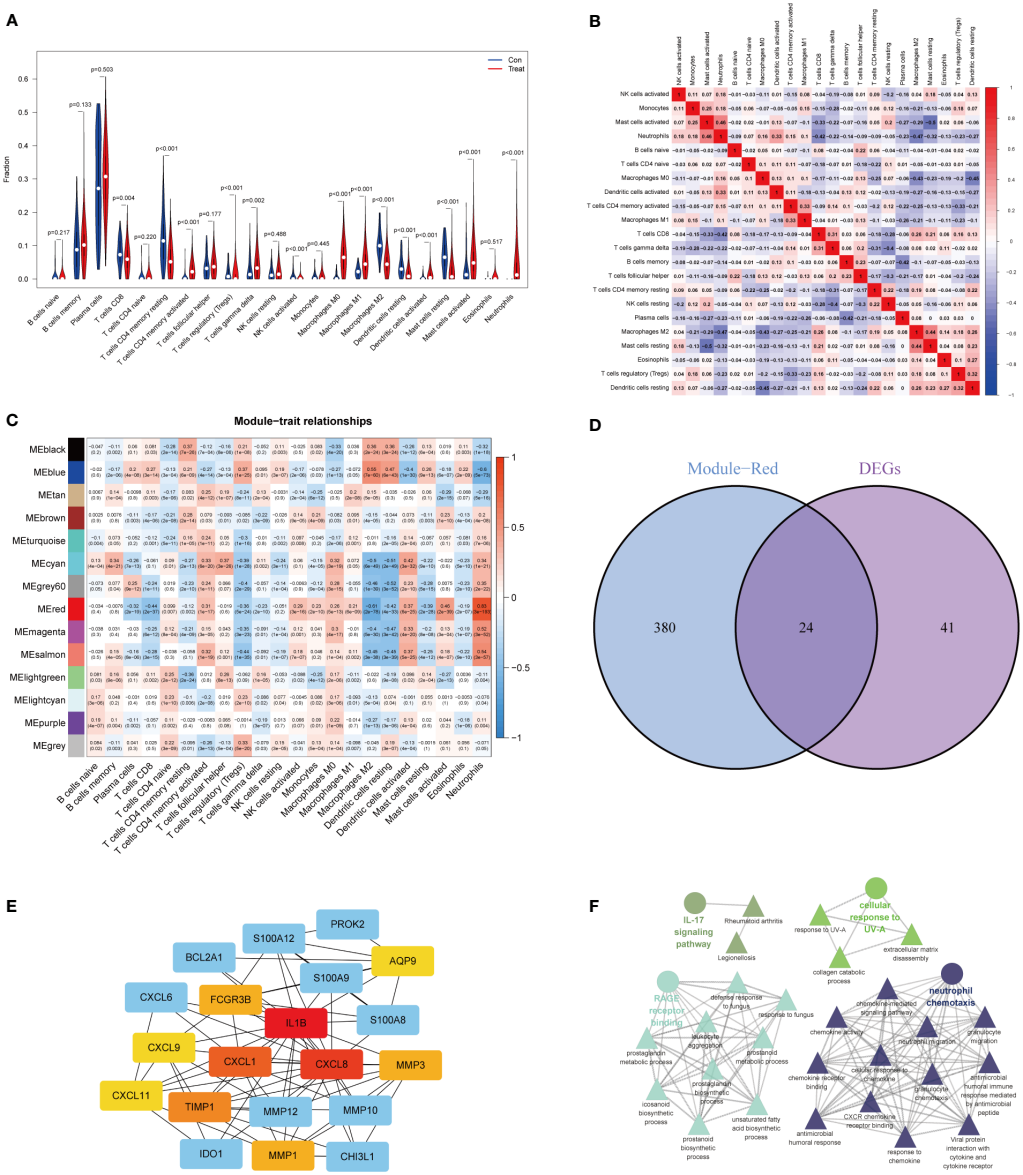
Identification of neutrophil-associated differentially expressed genes (NADEGs). **(A)** Comparison of significantly different immune cell infiltration in UC and healthy controls. **(B)** Heatmap of correlations of 22 immune cells in UC samples. Red indicated positive correlations and blue indicated negative correlations. **(C)** Heatmap of the correlation between immune cell on module genes by WGCNA. **(D)** Venn diagram showed the neutrophil-associated differentially expressed genes. **(E)** Subnetwork of core genes from the PPI network. Node color reflected the degree of connectivity (red color represented a higher degree, and blue color represented a lower degree). **(F)** Interaction pathway between NADEGs by Cluego. The circles represented the main enrichment pathways.

Based on CIBERSORT estimates, we determined the relative infiltration abundance of immune cell types in UC and healthy control samples. The majority of immune cells infiltrating the UC were more abundant than those in the control (**Figure 2A** and **Table S4**). In UC samples, neutrophils, mast cells, activated CD4 T cells, T cell gamma delta, M0 macrophages, and M1 macrophages were the major infiltrating immune cells, suggesting the involvement of multiple immune cells. **Figure 2B** showed the correlation between immune cells. Next, we used the WGCNA package to construct co-expression networks and calculated average linkages and Pearson correlation values to cluster the samples. The logarithm of node connectivity K (K) was negatively correlated with the logarithm of node probabilities (P(K)) with a correlation coefficient above 0.85. Based on the gene expression matrix and the proportion of immune cell types, the optimal soft threshold power was 15 (**Figure S2**). We created a hierarchical clustering tree using a dynamic hybrid cut method, where genes with similar expressions were tightly grouped to form a branch representing a gene module. Different branches of this tree represent different gene modules, with high co-expression levels of genes within the modules. As a result, 14 main modules were identified (**Figure 2C**). In order to select important modules to research the relationship between modules and immune cells, we found that the red module had the strongest correlation with neutrophils (R=0.83, P=3e-193) based on Pearson correlation coefficients between modules and sample characteristics. The infiltration characteristics of neutrophils in UC were further explored in combination with the enrichment results of DEGs. Therefore, 24 neutrophil-associated differentially expressed genes (NADEGs) were obtained by focusing on the red module and intersecting the genes in the red module with DEGs (**Figure 2D**). The core genes in the NADEGs were then identified using the CytoHubba plugin in Cytoscape. Using the Degree algorithm, we identified the top 5 hub genes as IL1B, CXCL8, CXCL1, TIMP1, and FCGR3B (**Figure 2E**). ClueGO then analyzed the NADEGs for pathway and process enrichment. RAGE receptor binding, cellular response to UV-A, IL-17 signaling pathway, and neutrophil chemotaxis were highly enriched (**Figure 2F**).

### Two distinct subtypes of neutrophil infiltration identified

**Figure 3:**
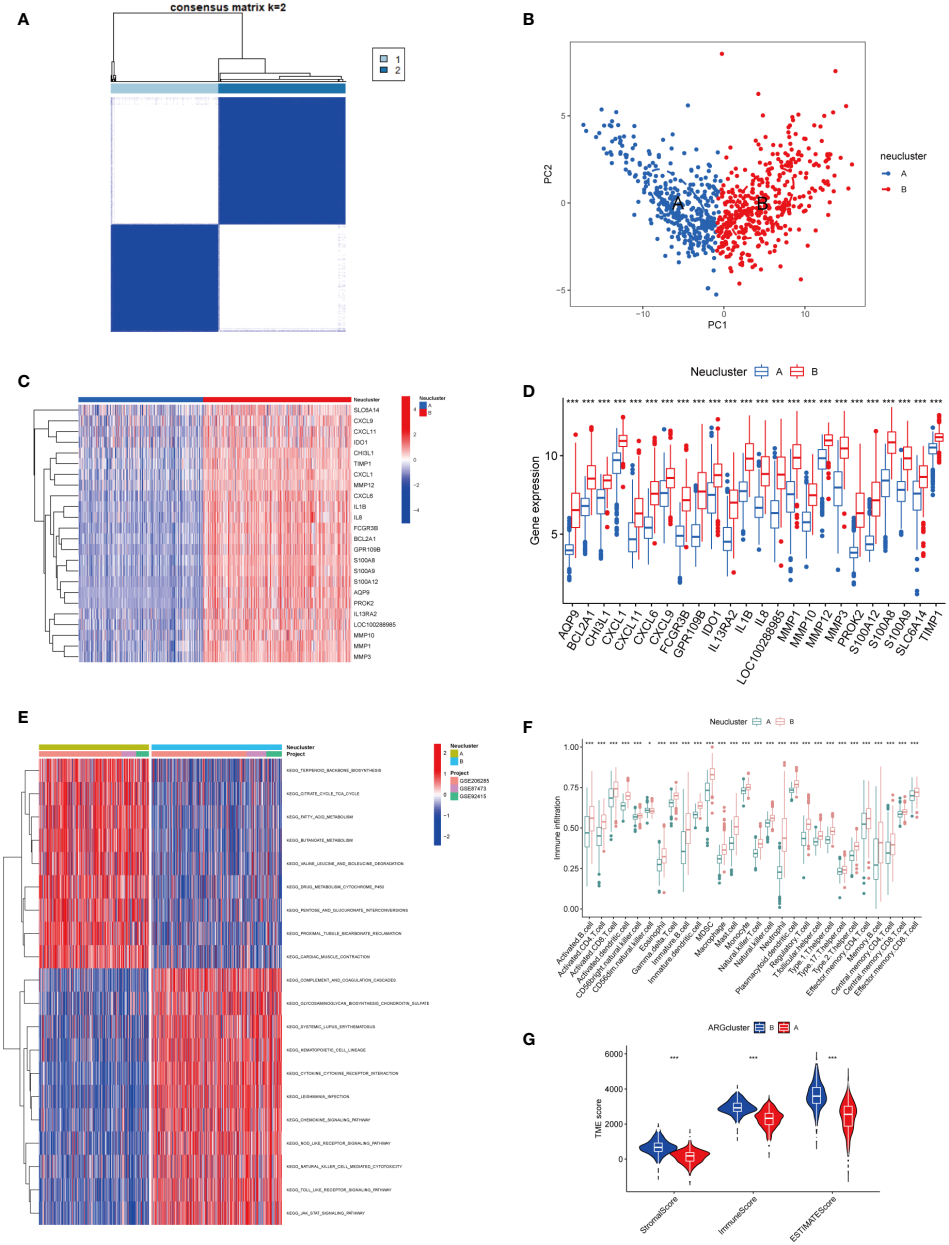
Exploration of neutrophil-associated subtypes and biological features. **(A)** Consensus matrix heatmap defining two clusters (k = 2) and their correlation area. **(B)** PCA analysis showed significant differences in transcriptomes between the two subtypes. **(C, D)** Heatmap and boxplot of the expression of 24 NADEGs in different clusters. Red for upper regulation, blue for lower regulation. **(E)** GSVA of biological pathways between two distinct subtypes, in which red represented activated and blue inhibited pathways. **(F)** The ssGESA results indicated the abundance of 28 infiltrating immune cells in different subtypes. The asterisks represented the statistical P-value (*P < 0.05; **P < 0.01; ***P < 0.001). **(G)** Correlations between the two subtypes and TME score. The asterisks represented the statistical P-value (*P < 0.05; **P < 0.01; ***P < 0.001).

Using the ConsensusClusterPlus package in R software, different neutrophil infiltration subtypes were identified using consensus clustering based on 24 NADEGs, and two clusters (cluster A and cluster B) were identified (**Figure 3A**). There were 403 samples in cluster A and 340 samples in cluster B. PCA showed that NADEGs could completely distinguish between the two subtypes (**Figure 3B**). Then, the heatmap and boxplot plot illustrated the differential expression levels of the 24 NADEGs between the two clusters (**Figures 3C, D**). The expression levels in cluster A were all lower than those in cluster B. GSVA enrichment analysis was then conducted to understand the biological changes behind the different clusters. **Figure 3E** and **Table S5** showed that subtype B is significantly enriched in immune-related pathways such as complement and coagulation cascades, cytokine-cytokine receptor interaction, chemokine signaling pathway, NOD-like receptor signaling pathway, natural killer cell-mediated cytotoxicity, TOLL like receptor signaling pathway, and JAK-STAT signaling pathway. Next, we compared the differences in immune cell infiltration between the two clusters using the ssGSEA method (**Table S6**). In **Figure 3F**, there was a higher infiltration of immune cells, especially neutrophils and various types of macrophages, in cluster B compared to cluster A. The ESTIMATE package was also used to assess the microenvironment score (immune score, stromal score, and estimated score) for both subtypes (**Figure 3G**).

### Neutrophils infiltration might affect the conversion of UC to CAC

**Figure 4:**
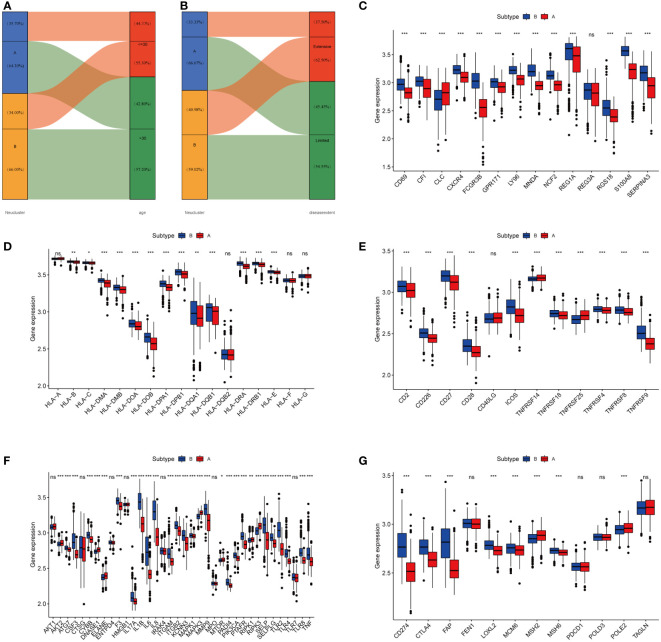
High-infiltration neutrophils of subtype B were more likely to develop CAC. **(A, B)** Alluvial diagram of subtype distributions in groups with different clinical features. **(C)** Gene expression of CAC-related gene between two distinct clusters. **(D, E)** Gene expression of HLA and MHC gene sets between two distinct clusters. **(F)** Gene expression of NETs-related gene sets between two distinct clusters. **(G)** Expression of immune checkpoints between two different clusters. The asterisks represented the statistical P-value (ns, not significant; P > 0.05; *P < 0.05; **P < 0.01; ***P < 0.001).

Age and disease extent are risk factors for colitis-association colorectal cancer, and patients with UC who are younger and have more extensive lesions are more likely to convert to CAC (29, 30). We observed a different distribution of age and extent of disease between the two subtypes. As shown in **Figure 4A**, a greater proportion of patients in cluster A were over 30 years of age (64.30%), while more than half of the patients under 30 years of age were from cluster B (55.30%). In terms of the extent of the disease, more patients with extensive lesions were from cluster B (62.50%), while 66.67% of patients in cluster A presented with limited lesions (**Figure 4B**). Following that, we examined the GSE37283 dataset for DEGs between UC and CAC. Using the LIMMA package, we set |LogFC>2|, p<0.05 to identify 14 genes associated with CAC (**Figure S3**). We next compared the differences in the expression of the CAC-associated genes between different neutrophil subtypes. We found that most CAC-associated genes were highly expressed in cluster B, and therefore we concluded that cluster B with highly infiltrated neutrophils was more likely to transform from UC to CAC (**Figure 4C**). The major histocompatibility complex and T-cell stimulating factor were investigated concerning the two subtypes. Major histocompatibility complex and T-cell stimulating factor expression levels were higher in cluster B, but not in TNFRSF14 and TNFRSF25 (**Figures 4D, E**). Recent studies have shown that highly infiltrated neutrophils in UC could form neutrophil extracellular traps that contribute to the transformation of colitis into cancer (24). From the review, we collected mostly information on ligands and receptors that promote the NET formation, downstream signals, and molecules identified as NET adhesion frameworks (31). According to our results (**Figure 4F**), almost genes related to neutrophil extracellular traps were highly expressed in subtype B, suggesting that more neutrophil extracellular traps might be present in cluster B (32). Furthermore, we compared the differential expression of immune checkpoints between the two subtypes, and we found that most immune checkpoints, including PD-L1 and CTLA4, were highly expressed in subtype B (**Figure 4G**).

### Identification of valid diagnostic markers of inflammatory-cancer conversion

**Figure 5:**
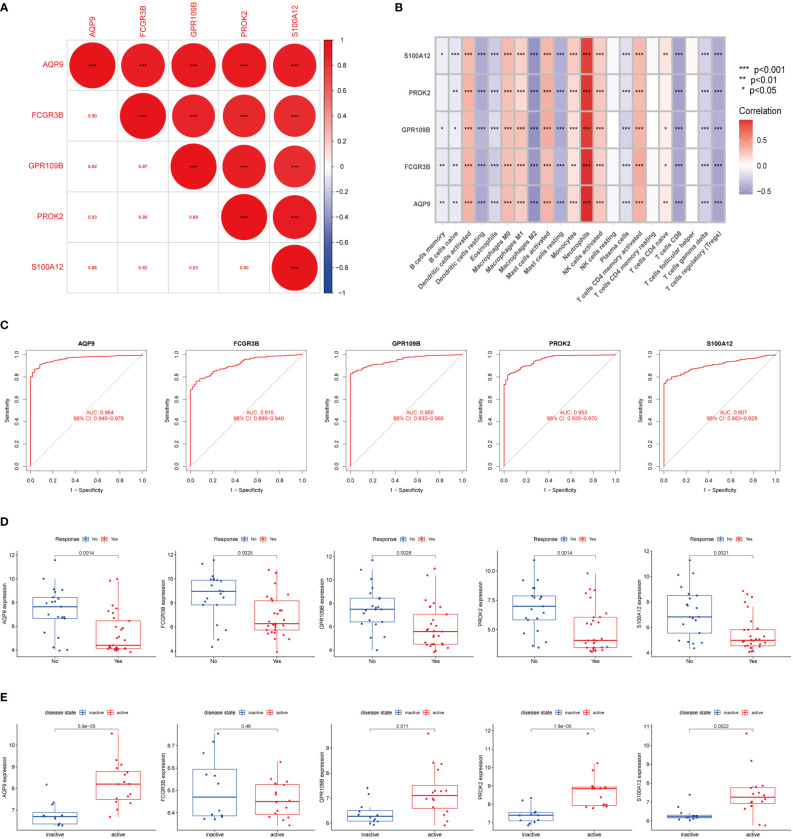
Identification of DEGs between subtypes **(A)** The correlation of five DEGs. Red represented positive correlation. **(B)** Pearson correlation analysis revealed that immune cells had strong positive and negative correlations with DEGs. Red represented positive correlation, purple represented negative correlation. **(C)** The area under the ROC curve indicates the effectiveness of the five DEGs in the diagnosis of UC. **(D)** Boxplot indicated that 5 DEGs could predict the effect of anti-TNF-α treatment in GSE92415 dataset. **(E)** Boxplot showed that in the GSE53306 cohort the expression of five DEGs could distinguish between active UC and remission UC, except for FCGR3B. The upper and lower ends of the boxes represented the interquartile range of values. The lines in the boxes represented the median value. The asterisks represented the statistical p-value (*P < 0.05; **P < 0.01; ***P < 0.001).

To better find genes that could diagnose UC or even CAC, we identified 5 DEGs (AQP9, FCGR3B, GPR109B, PROK2, S100A12) between neutrophil-associated subtypes using the LIMMA package. Firstly, we examined the correlation between these 5 genes, which was highly correlated as illustrated in **Figure 5A**. Next, we accessed the correlations between these genes and immune cells. These genes were also correlated with other immune cells besides neutrophils (**Figure 5B**). To account for the ability of each gene to distinguish between UC and normal samples, the area under the ROC curve (AUC) was calculated to assess the diagnostic value of these genes (**Figure 5C**). Next, we compared the expression of 5 genes in the GSE92415 and GSE16879 cohort receiving anti-TNF-α treatment to determine whether the effect of anti-TNF-α treatment could be assessed. As shown in **Figure 5D** and **Figure S4**, the expression levels of all 5 genes were decreased in the response-yes group, suggesting that these genes could also be used as markers to assess the response of anti-TNF-α treatment. Furthermore, we investigated whether these five genes could be used to distinguish between patients with active UC and those with remission UC in the GSE53306 cohort. Apart from FCGR3B, the expression of the remaining genes was higher in active UC, as shown in **Figure 5E**.

### Differential expression of markers in mouse models

**Figure 6:**
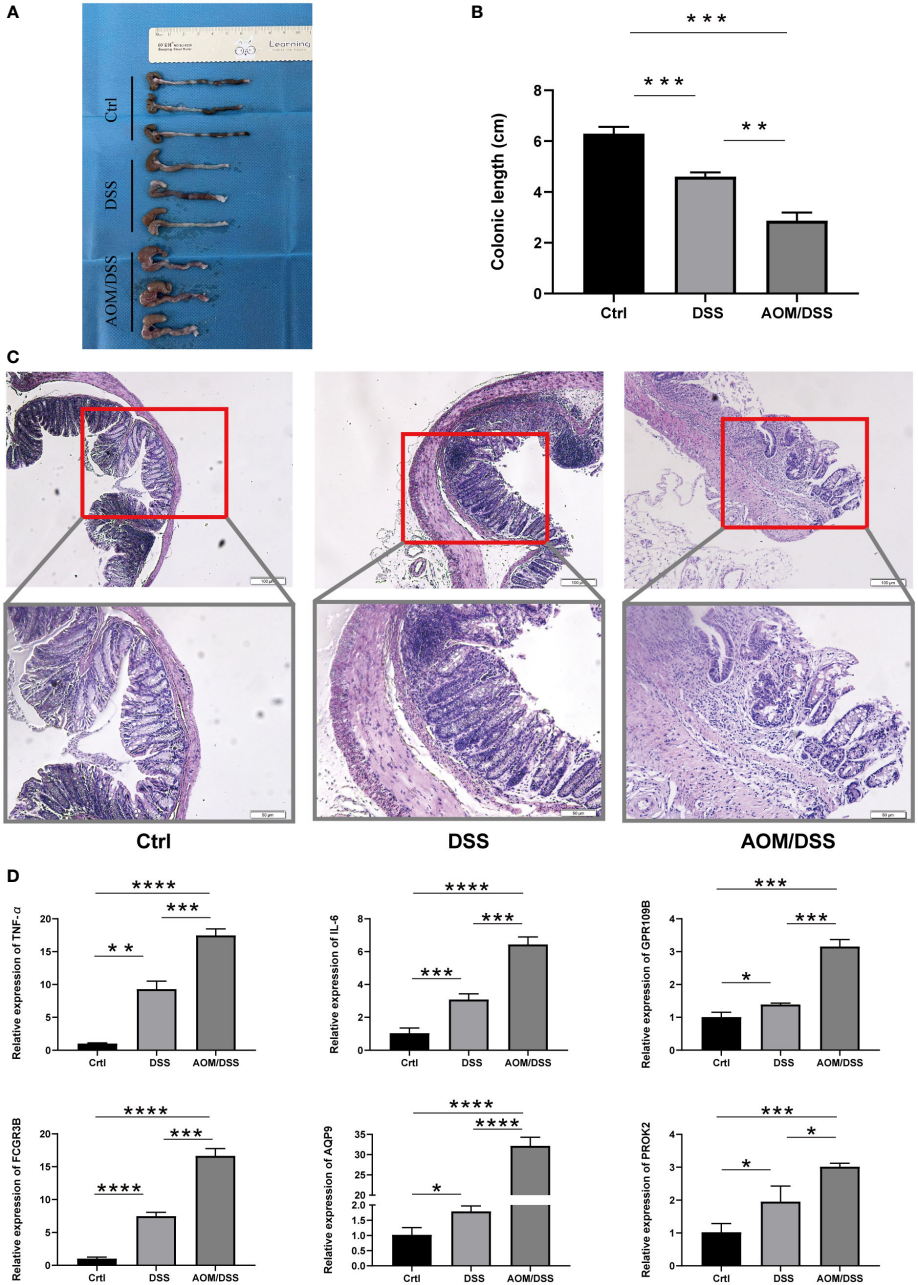
Expression of five markers in the mouse model. **(A, B)** Colon length of different groups of mice. **(C)** Representative H&E staining of different groups of **(D)** colon. **(C)** RT-PCR analysis of differential gene expression results in the Crtl, DSS and AOM/DSS groups. The asterisks represented the statistical p-value (*P < 0.05, **P < 0.01, ***P < 0.001, and ****P < 0.0001).

DSS-induced colitis and AOM/DSS-induced CAC were used to further validate the expression of biomarker genes. The colonic length of mice with different models exhibited significant differences from control mice (**Figures 6A, B**). Histological analysis indicated the presence of damage to the intestinal epithelium in mice with enterocolitis, and mice in the AOM/DSS group had significant dysplasia (**Figure 6C**). We also examined the expression levels of the IL-6, TNF-α, and biomarker genes using RT-qPCR. Gene expression was increased in the DSS group compared to the Ctrl group and was even more significantly upregulated in the mice with AOM/DSS group (**Figure 6D**).

### AOM/DSS mice recruit more neutrophils

**Figure 7:**
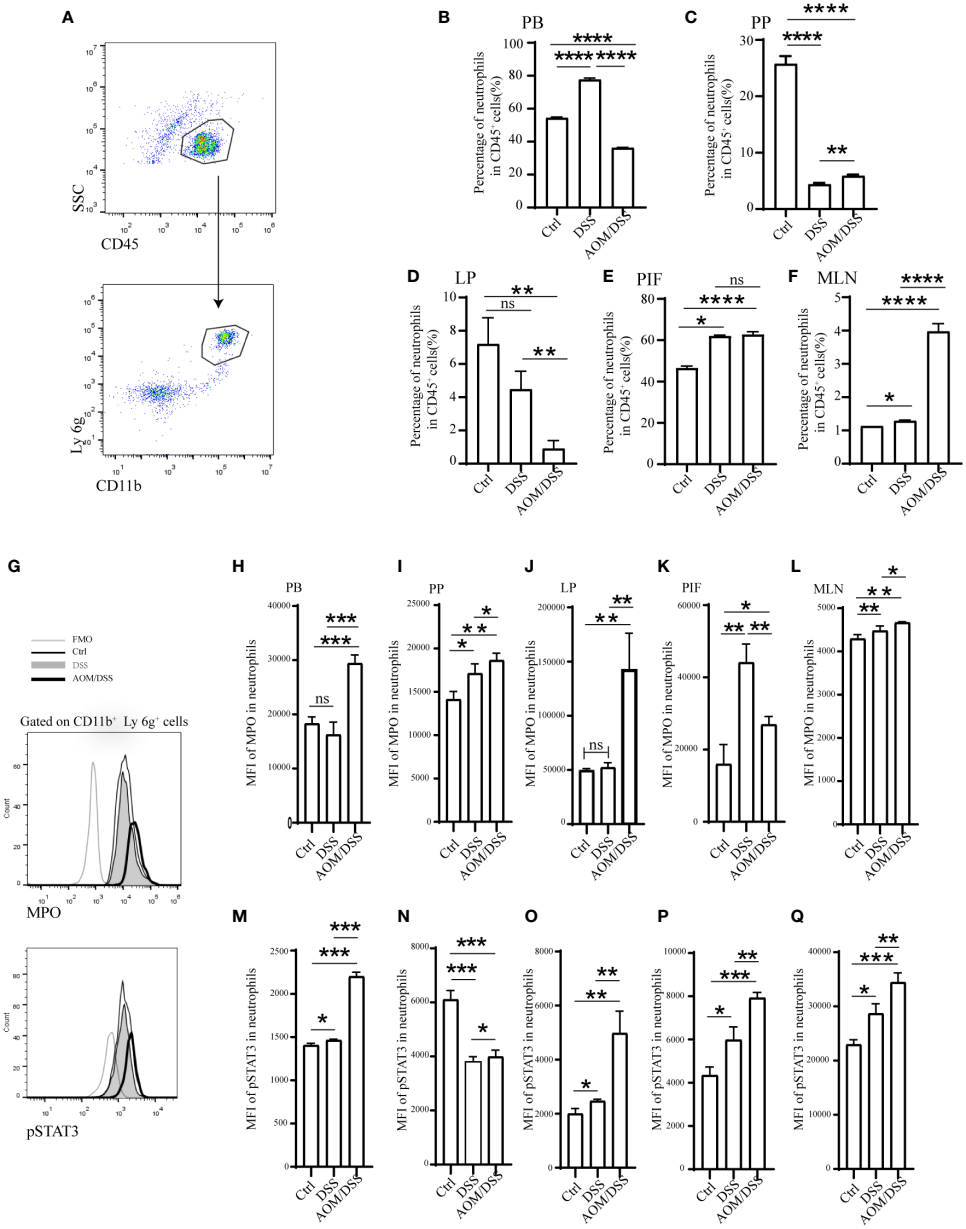
Enhanced STAT3 phosphorylation and MPO secretion in expanded neutrophils of mice were periodically treated with DSS or AOM/DSS. Then, mice were sacrificed to obtain PB, PP, LP, MLN, and PIF. The percentage of neutrophils was detected after Ficoll-Hypaque separation and lysed red blood cell. **(A, G)** Flow-cytometry gating strategy for neutrophils, MPO and pSTAT3. Percentage of neutrophils analyzed by flow-cytometry in the PB **(B)**, PP **(C)**, LP **(D)**, PIF **(E)**, and MLN **(F)**. Mean fluorescence intensity of MPO in the PB **(H)**, PP **(I)**, LP **(J)**, PIF **(K)**, and MLN **(L)**. Mean fluorescence intensity of pSTAT3 in the PB **(M)**, PP **(N)**, LP **(O)**, PIF **(P)**, and MLN **(Q)**. PB, peripheral blood; PP, peyer’s patch; LP, lamina propria; MLN, mesenteric lymph node; PIF, peritoneal irrigation fluid; MFO, fluorescence minus one. *P<0.05; **P< 0.01, ***P<0.001, ****P<0.0001. ns, not significant.

Then we proceeded to further investigate the variation in neutrophil infiltration with different mouse models (Control, DSS, and AOM/DSS). The initiation of colitis resulted in a mass recruitment of CD11b+ Ly 6g+ neutrophils in PB, MLN, and PIF (**Figures 7A, B, E, F**, and **Figure S5**). The proportion of neutrophils in PP of mice with AOM/DSS was higher than that of DSS (**Figure 7C**) but declined in LP (**Figure 7D**). Next, we compared the expression of MPO in neutrophils by flow analysis to compare the differences between the different groups (33, 34). In addition, DSS mice showed an increased level of MPO in PB, PP, LP, MLN, and PIF (**Figures 7G–L**), and even higher in AOM/DSS mice (**Figures 7H–J, L**). JAK-STAT signaling pathway has been demonstrated to play a potential role in inflammation and tumor development. The elevated levels of pSTAT3 in PB, LP, MLN and PIF of AOM/DSS mice (**Figures 7M, O–Q**) were consistent with the results of GSVA (**Figure 3E**). However, for DSS mice, the pSTAT3 levels in PP did not change significantly (**Figure 7N**). These results established an increased percentage of neutrophils and MPO, pSTAT3 expression in DSS and AOM/DSS mouse models. These results also indicated that the neutrophils and the JAK-STAT pathway might be involved in the transformation of colitis to colitis-associated colorectal cancer.

## Discussion

The present study demonstrated that increased neutrophils in UC promote colitis progression and even the transformation to CAC. We found 65 DEGs between 734 samples from patients with UC and 60 samples from healthy controls. UC was associated with multiple immune response pathways, according to the GSEA results. According to CIBERSORT analysis, neutrophils were significantly increased in UC patients’ colonic tissue. Comprehensive bioinformatics analysis and *in vivo* validation using the classical AOM/DSS mouse model identified AQP9, FCGR3B, GPR109B, PROK2, and S100A12 as markers for the diagnosis and determination of UC progression to CAC. Through flow cytometry analysis, we found a positive correlation between neutrophils and UC severity, providing new insight into how UC transforms into CAC.

Chronic mucosal inflammation is the hallmark of inflammatory bowel disease (IBD), especially ulcerative colitis. Long-term chronic mucosal inflammation has long been known to be a contributing factor in the development of cancer (35). Unlike sporadic colorectal cancer, CAC begins in the swollen epithelium and progresses in an “inflammation-dysplasia-cancer” pathway (11). A chronic inflammation-induced signaling pathway might promote carcinogenesis by enhancing oxidative stress, epithelial cell proliferation, and neovascularization (11, 35, 36). Toll-like receptors (TLR), JAK/STAT signaling pathway, nuclear factor κB (NF-κB), mammalian target of rapamycin complex (mTOR), autophagy, and oxidative stress have been suggested as molecular mechanisms present in the progression of CAC (37). Numerous studies have shown that in IBD, especially the JAK/STAT pathway interferes with many inflammatory pathways (38). STAT3 has been documented as the most important member of the JAK/STAT family in IBD. Phosphorylated STAT3 was found in mucosal T cells of IBD patients with high STAT3 activity and expression (39, 40). The IL6-STAT3 signaling pathway in T cells can promote the survival of pathogenic T cells (41). In our results, JAK/STAT pathways were greatly enriched in subtype B of high neutrophil infiltration. In addition, IL-6 expression was significantly increased in the AOM/DSS mouse model compared to the control and DSS groups. pSTAT3 expression in PB, LP, MLN, and PIF also showed significantly higher expression levels in the AOM/DSS simulated CAC disease model. These results suggest that the JAK-STAT pathway might play an important role in the progression of colitis to cancer.

Numerous pathologies are associated with neutrophil-driven inflammation (42, 43). As one of the most abundantly expressed proteins in neutrophils, MPO is recognized as a marker of neutrophil pathological function in neutrophils and disease, and its release into the extracellular environment might cause tissue damage and thereby exacerbate inflammation. Plasma MPO levels are often elevated in patients and correlate with disease severity (44–46). In addition, neutrophils could release NETs to promote the production of pro-inflammatory mediators by other cells, dysfunction the intestinal epithelial barrier, denature the extracellular matrix, and maintain the malignant inflammatory cycle of IBD (47). In our study, neutrophils infiltrated with more in cluster B, we found more expression of ligands and receptors that stimulate the formation of NETs, downstream-related signals, and NETs framework molecules. In our mouse model, the expression of MPO was increased in neutrophils from DSS mice. The expression of MPO was higher in the AOM/DSS group compared to the DSS group. These findings suggest a potential role of neutrophils in the transformation of colitis to intestinal cancer.

We finally identified 5 biomarkers for the diagnosis of CAC. ACP9 could regulate the synthesis and function of mucus and tissue-specific physiological properties in UC tight junctions (48). S100A12 is a calcium-binding protein found specifically in granulocytes. It belongs to the S100 family of calcium-binding proteins. Neutrophil-derived S100A12 is strongly upregulated during active IBD, and increased serum and fecal S100A12 protein concentrations are associated with disease activity (49, 50). Prokineticin 2 (PROK2) is an inflammatory cytokine-like molecule expressed primarily by macrophages and neutrophils infiltrating damaged tissues. PROK2 might serve as a potential biological marker for UC (51). FCGR3B is a stimulatory Fcγ receptor that promotes neutrophil recruitment and immune complex capture and clearance, and deletion of FCGR3B results in immune complex-mediated disease (52). In addition, it has been suggested that FCGR3B may contribute to UC pathogenesis by increasing the copy number of the gene (53). In neutrophils, GPR109B is abundantly expressed, and its activation induces chemotaxis (54). According to these findings, these biomarkers are associated with neutrophils and accurately predict CAC development.

In conclusion, we demonstrated by bioinformatics that UC patients with more neutrophil infiltration are more prone to CAC and have higher expression of ligands and receptors that stimulate NETs formation, downstream related signals, and molecules linked to the NETs framework. The JAK/STAT pathway was also significantly enriched in subtype with high neutrophil infiltration. In the DSS-induced colitis model and the AOM/DSS-induced CAC model, neutrophil infiltration increased with disease progression. Among them, MPO expression was also increased in neutrophils. In neutrophils, we also observed increased pSTAT3 expression. This suggests that neutrophils and the JAK/STAT pathway might promote the transformation of colitis to intestinal cancer. Although bioinformatic methods suggested that NET formation contributed to the conversion of UC to CAC, more detailed studies are necessary. Moreover, advanced methods like single-cell sequencing will provide a better understanding of how neutrophils influence the transformation of colitis into intestinal cancer, as well as reveal new therapeutic targets for disease prediction and treatment.

## Data availability statement

The datasets analyzed for this study can be found in the GEO (https://www.ncbi.nlm.nih.gov/geo/query/acc.cgi?acc=GSE87473, GSE92415, GSE206285, GSE87473, GSE53306, GSE16879, and GSE37283).

## Ethics statement

The animal study was reviewed and approved by The Animal Health Research Committee of the First Hospital of Jilin University.

## Author contributions

CZ, ZM, and HY conceived the project. CZ, YZ, ZS, and JB contributed to data acquisition, analysis and interpretation, and manuscript writing. CZ, ZM, and JZ conducted the experiments and revised the manuscript. All authors contributed to the article and approved the submitted version.
