# Corrigendum: Identifying neutrophil-associated subtypes in ulcerative colitis and confirming neutrophils promote colitis-associated colorectal cancer

**DOI:** 10.3389/fimmu.2024.1376797

**Published:** 2024-04-10

**Authors:** Chen Zhang, Jiantao Zhang, Yanli Zhang, Zian Song, Jing Bian, Huanfa Yi, Zhanchuan Ma

**Affiliations:** ^1^ Colorectal & Anal Surgery Department, General Surgery Center, First Hospital of Jilin University, Changchun, Jilin, China; ^2^ Central Laboratory, First Hospital of Jilin University, Changchun, Jilin, China; ^3^ Echocardiography Department, First Hospital of Jilin University, Changchun, Jilin, China; ^4^ Department of Respiratory Medicine, First Affiliated Hospital of Jilin University, Changchun, Jilin, China

**Keywords:** ulcerative colitis, colitis-associated colorectal cancer, immune cell infiltration, biomarker, neutrophil

In the published article, there was an error. Detailed sample information was missing.

A correction has been made to **Materials and methods**, *Data collection and processing*, paragraph 1 This sentence previously stated:

“A total of 803 RNA-seq samples were downloaded from datasets GSE87473, GSE92415, and GSE206285, including untreated 743 UC samples and 60 healthy control samples”

The corrected sentence appears below:

“A total of 803 RNA-seq samples were downloaded from datasets GSE87473, GSE92415, and GSE206285, including untreated 743 UC samples and 60 healthy control samples. It should be noticed that though 364 patients with UC were treated with ustekinumab (data extracted from database GSE206285), RNA extraction and microarray analysis of biopsy samples collected when the data set was uploaded at baseline. Baseline is the period of time in a clinical study when a patient has been screened to for the study but has not yet started medication.”

The authors apologize for this error and state that this does not change the scientific conclusions of the article in any way. The original article has been updated.

