# Supplementary material for: Identifying neutrophil-associated subtypes in ulcerative colitis and confirming neutrophils promote colitis-associated colorectal cancer
Source: Front Immunol. 2023 Feb 10;14:1095098. doi: 10.3389/fimmu.2023.1095098 (PMC9950623; doi:10.3389/fimmu.2023.1095098)
Supplement: Supplementary file 1 [file DataSheet_1.docx]

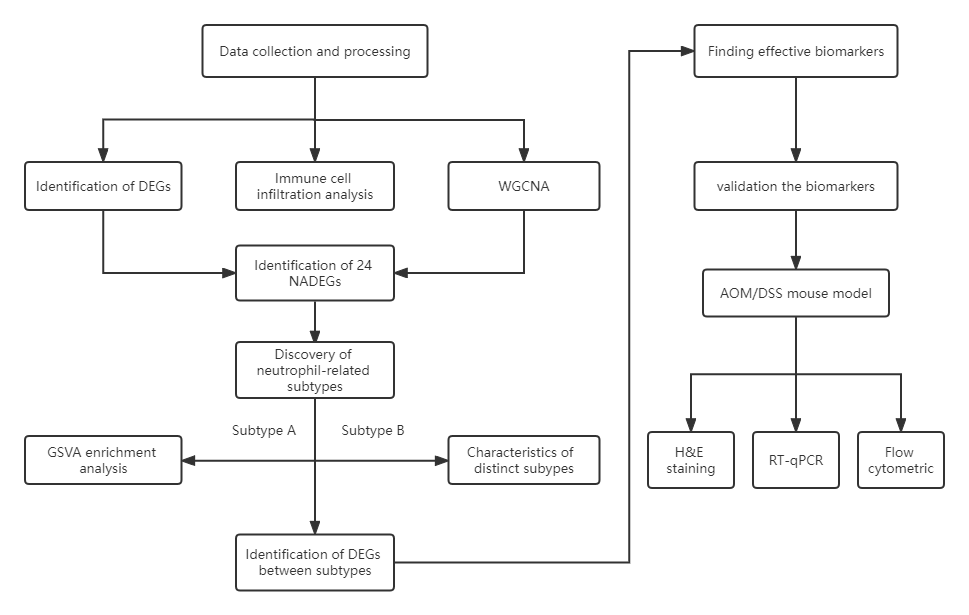


**Figure S1** The whole analytical process of the study.


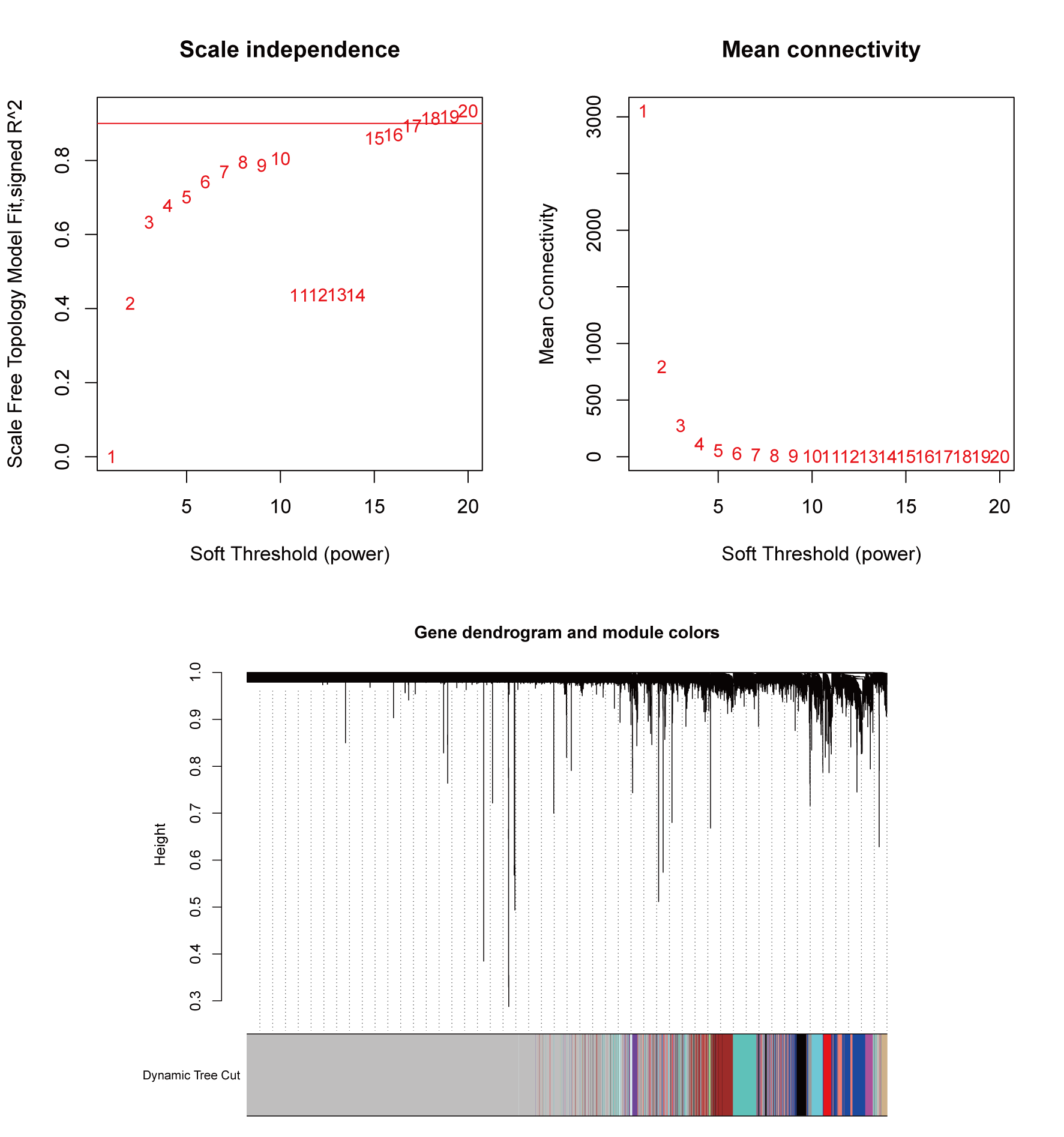


**Figure S2** The scale-free fit index and mean connectivity for various soft-thresholding powers, and module identification based on the dynamic pruning tree.


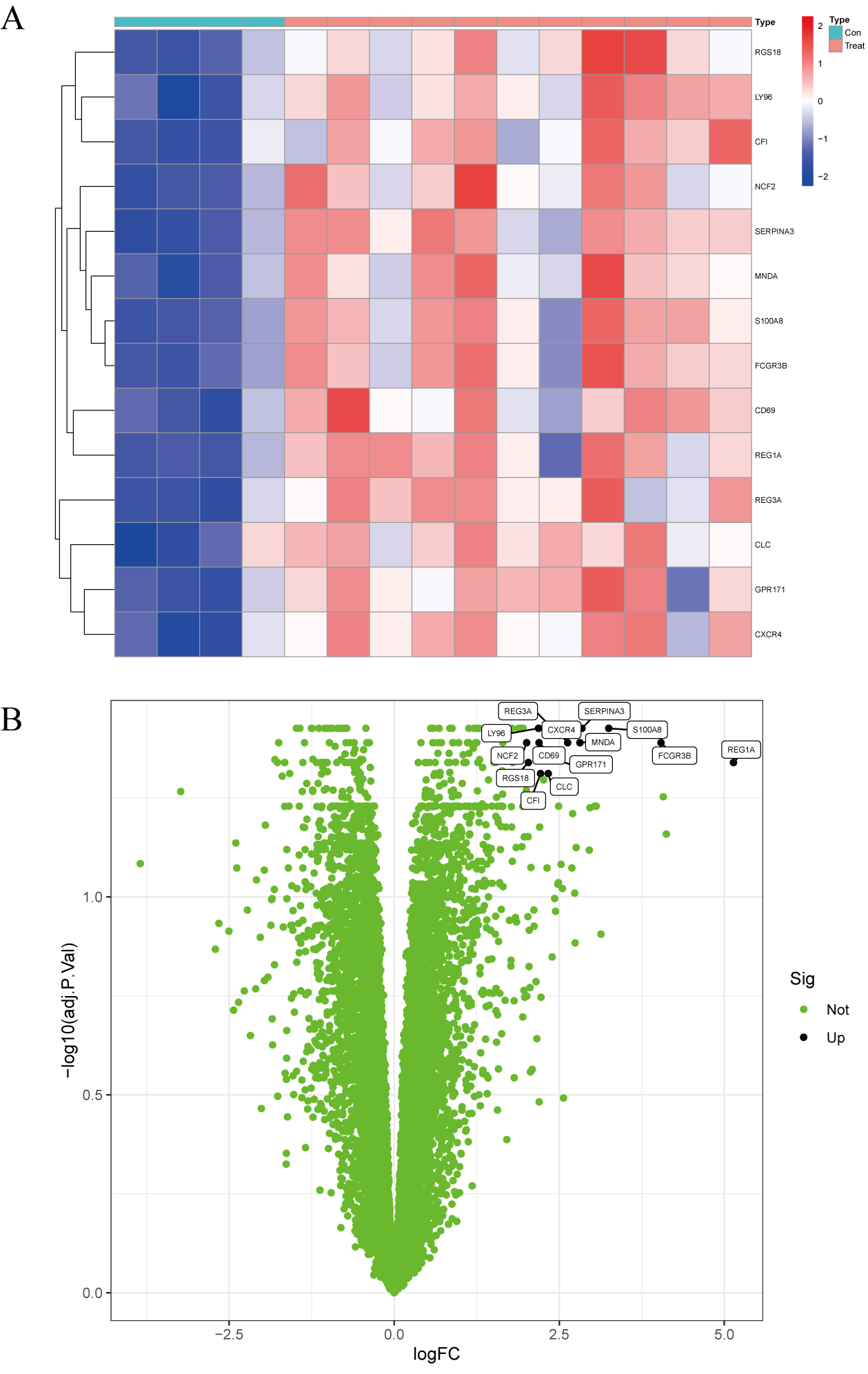


**Figure S3** (A-B) Heatmap and volcano map showed 14 differentially expressed genes between UC and CAC in dataset GSE37283.


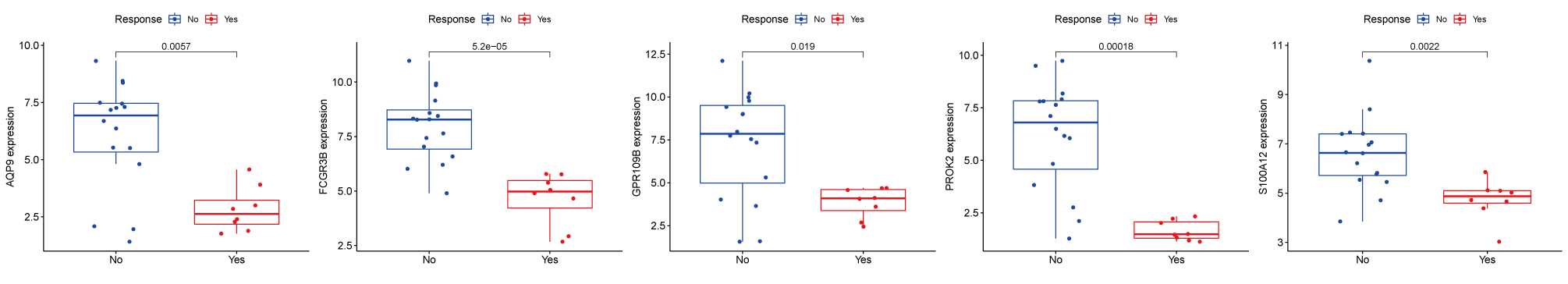


**Figure S4** Boxplot indicated that 5 DEGs could predict the effect of anti-TNF-α treatment in the GSE16879 dataset.


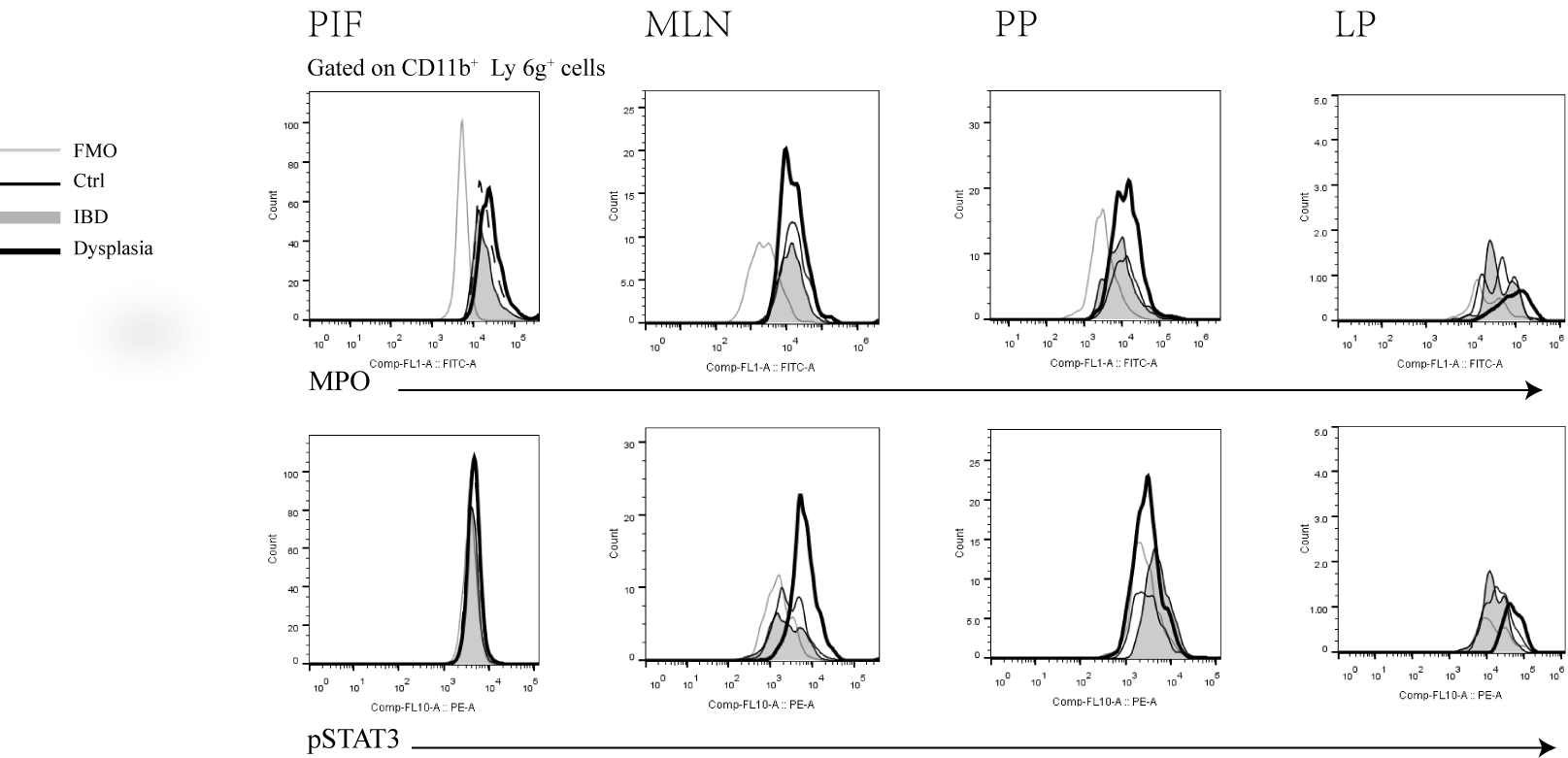


**Figure S5** Expression of STAT3 phosphorylation and MPO in expanded neutrophils of IBD. 8-10 weeks mice with dysplasia in the colon were sacrificed to obtain PB, PP, LP, MLN, and PIF. The expression level of pSTAT3 and MPO in neutrophils (PIF, MLN, PP, LP) were detected by flow cytometry after Ficoll-Hypaque separation and lysed red blood cells.

PB, peripheral blood; PP, peyer’s patch; LP, lamina propria; MLN, mesenteric lymph node.
